# Bone management in Japanese patients with prostate cancer: hormonal therapy leads to an increase in the FRAX score

**DOI:** 10.1186/s12894-016-0151-9

**Published:** 2016-06-17

**Authors:** Takashi Kawahara, Shusei Fusayasu, Koji Izumi, Yumiko Yokomizo, Hiroki Ito, Yusuke Ito, Kayo Kurita, Kazuhiro Furuya, Hisashi Hasumi, Narihiko Hayashi, Yasuhide Myoshi, Hiroshi Miyamoto, Masahiro Yao, Hiroji Uemura

**Affiliations:** Department of Urology, Yokohama City University, Graduate School of Medicine, Yokohama, Japan; Department of Urology and Renal Transplantation, Yokohama City University Medical Center, 4-57 Urafune-cho, Minami-ku, Yokohama, Kanagawa 232-0024 Japan; Departments of Pathology and Urology, Johns Hopkins University School of Medicine, Baltimore, USA

**Keywords:** Androgen deprivation therapy, FRAX, Prostate cancer, Bone fracture

## Abstract

**Background:**

Osteoporosis is a common consequence of androgen deprivation therapy (ADT) for prostate cancer. Up to 20 % of men on ADT have suffered from fractures within 5 years. The WHO Fracture Risk Assessment Tool (FRAX) has been utilized to predict the 10-year probability of major osteoporotic and hip fracture. However, to date, no large studies assessing the utility of the FRAX score in prostate cancer patients with or without ADT have been performed. We herein evaluated the impact of ADT on the FRAX score in prostate cancer patients.

**Methods:**

The assessment of the FRAX score was performed in a total of 1220 prostate cancer patients, including patients who underwent brachytherapy (*n* = 547), radical prostatectomy (*n* = 200), external beam radiation therapy (*n* = 264) and hormonal therapy alone (*n* = 187) at Yokohama City University Hospital (Yokohama, Japan). We evaluated the effect of ADT on the FRAX score.

**Results:**

Using the FRAX model, the median and mean 10-year probability of a major osteoporotic fracture according to the clinical risk factors alone was 7.9 % (8.8 ± 4.3 %), while the 10-year probability of hip fracture risk was 2.7 % (3.5 ± 3.1 %). In the ADT group, the duration of ADT was correlated with both major osteoporotic risk and hip fracture risk (R^2^ = 0.141, *p* < 0.001 and R^2^ = 0.166, *p* < 0.001, respectively). A comparison between the ADT (*n* = 187) and non-ADT (*n* = 399) groups demonstrated that the major fracture risk was > 20 % higher and the hip fracture risk was > 3 % higher in the ADT group than in the non-ADT group (ADT: 10 (5.3 %) and 118 (63.1 %), non-ADT 13 (3.3 %) and 189 (47.4 %), *p* < 0.001 and *p* < 0.001, respectively).

**Conclusions:**

These results suggested that the longer duration of ADT led to an increased FRAX score, and the FRAX score may be a predictor of bone management treatment, particularly in prostate cancer patients.

## Background

Following the widespread implementation of PSA screening, the incidence of prostate cancer has been increasing in Japan [1, 2]. By the end of 2015, 98,400 men will have been newly diagnosed and 12,200 men will have died of prostate cancer. Many patients diagnosed with prostate cancer are elderly and must be treated with hormonal therapy [3]. Men with prostate cancer are often at risk for other age-related adverse events, such as hip fractures. The risk of hip fractures can increase in men with prostate cancer because the bone density often decreases due to androgen deprivation therapy (ADT), occult bone metastases, or a combination thereof [4–8]. The mechanism is believed to be due to a decrease in sexual hormone levels, which induces receptor activator of nuclear factor-kappa-B ligand (RANKL) expression from osteoblasts. Consequently, osteoclasts are involved in bone resorption [9]. The skeleton is the third most common site of metastatic cancers, and one-third to one-half of all cancers metastasize to the bone. In prostate cancer, bone metastasis is the most common site for tumor development [10]. Osteoporosis or low bone mineral density (BMD) is a highly prevalent health problem in the elderly as well as in prostate cancer patients treated with ADT. The FRAX score is a fracture risk assessment tool developed by the World Health Organization (WHO) to predict the fracture risk of patients according to clinical risk factors alone or in combination with BMD at the femoral neck [11]. It is a computer-based algorithm which provides the 10-year probability of hip and major osteoporotic fractures (e.g., clinical spine, forearm, hip, or shoulder fracture) according to age, sex, body mass index, and clinical risk factors [12, 13]. There have been no proven methods for predicting pathologic fractures in patients with skeletal metastasis thus far [14].

The fracture risk varies depending on the geographic location and ethnicity, and the FRAX algorithm has been calibrated to account for this [11]. Algorithms are available for diverse ethnic groups, including Asians, based largely on data from Japan and China [13]. Despite its importance, there have been few studies which investigated the fracture risk among Asians with prostate cancer living in Japan [13]. The present study evaluates the risk of developing hip fractures in Japanese men treated at one institute for prostate cancer [4] by examining whether or not ADT could influence the FRAX score.

## Methods

### Patients

A total of 1220 patients were enrolled in this study. All patients were pathologically diagnosed with prostate cancer and followed-up at Yokohama City University Hospital (Yokohama, Japan) and the FRAX score was calculated once during prostate cancer therapy. Written informed consent was obtained from all patients and this study protocol was approved by the Institutional Review Board at Yokohama City University Hospital.

The study cohort comprised 547 (44.8 %) patients who received brachytherapy, 200 (16.4 %) who received radical prostatectomy, 267 (21.9 %) who received external beam radiation therapy (EBRT), 187 (15.3 %) who received ADT monotherapy, and 19 (1.6 %) who received active surveillance. Excluding the ADT monotherapy group, 615 (50.4 %) patients received ADT in combination with surgical or radiation therapy. (Table 1) In the present study, EBRT was performed as a high-dose intensity-modulated radiation therapy. Some of the patients in the hormonal therapy group had bone metastasis at the time of diagnosis. To exclude bias, men with castration-resistant prostate cancer were not included in the present study. Data from each subject were entered into the FRAX algorithm and 10-year fracture probabilities were calculated using the clinical risk factors (CRFs) alone. Due to the differences between the FRAX score alone or in combination with BMD, we excluded the FRAX score using the BMD measurement. BMD was measured using dual-energy X-ray absorptiometry of the femoral neck.

**Table 1:** Patients’ background

Variables	number (%)
Total	1220 (100.0 %)
Brachy Therapy	547 (44.8 %)
with neo-adjuvant HTx	336 (27.5 %)
without HTx	211 (17.3 %)
Total Prostatectomy	200 (16.4 %)
with adjuvant HTx	50 (4.1 %)
without HTx	150 (12.3 %)
EBRT	267 (21.9 %)
with HTx	229 (18.8 %)
without HTx	38 (3.1 %)
Hormonal Tx only	187 (15.3 %)
Active surveillance	19 (1.6 %)

The FRAX tool (available at http://www.shef.ac.uk/FRAX/tool.jsp?lang=en) was used to compute the probability of a major osteoporotic event and hip fracture. The date of birth, weight (kg), height (cm), and a yes or no response for various CRFs were entered into the FRAX questionnaire, and the fracture probability was calculated for each subject. Childhood fractures reported by the subjects were not considered to be fragility fractures.

### Statistical analysis

All continuous variables were expressed as the means ± SD. The numerical data were compared using Student’s *t*-test. The correlation between 148 variables was determined using Spearman’s correlation coefficient. A *p*-value 149 of 0.05 or less was considered to be statistically significant.

## Results

A total of 1220 men were recruited to participate in this study. Their demographic characteristics are presented in Table 2. There were no differences in terms of age, body weight, body height, previous fractures, parent fractured hip, current smoking, use of glucocorticoids, rheumatoid arthritis, secondary osteoporosis, and alcohol consumption of 3 or more units per day, which are risk factors for fracture that are included in the FRAX score (Table 2).

**Table 2:** Results in each therapy with prostate cancer patients

Number (%) or median (Mean ± SD)					
Variables	Total (1220)	Brachy Tx (547)	Prostatectomy (200)	EBRT (267)	Hormonal monotherapy (187)
Age	74 (73.3 ± 7.1)	73 (72.1 ± 6.4)	72.5 (72.1 ± 7.1)	75 (74.6 ± 6.5)	77 (76.3 ± 8.2)
Weight (kg)	64 (64.3 ± 8.9)	64 (64.6 ± 8.7)	63.6 (63.7 ± 8.3)	65 (65.1 ± 9.2)	63 (63.3 ± 9.9)
Height (cm)	165 (165.5 ± 6.0)	165 (165.7 ± 6.1)	165 (165.4 ± 5.8)	166 (165.7 ± 5.9)	165 (164.6 ± 6.0)
Previous fracture	255 (20.9 %)	111 (20.2 %)	40 (20.0 %)	63 (23.6 %)	35 (18.7 %)
Parent Fractured Hip	90 (7.4 %)	38 (6.9 %)	19 (8.5 %)	18 (6.7 %)	12 (6.4 %)
Current Smoking	138 (11.3 %)	71 (13.0 %)	26 (13.0 %)	22 (8.2 %)	16 (8.6 %)
Glucocorticoid	38 (3.1 %)	3 (0.5 %)	4 (2.0 %)	8 (3.0 %)	19 (10.2 %)
Rheumatoid arthritis	13 (1.1 %)	3 (0.5 %)	3 (1.5 %)	3 (1.1 %)	4 (2.1 %)
Secondary osteoporosis	108 (8.9 %)	45 (8.2 %)	20 (10.0 %)	26 (9.7 %)	18 (9.6 %)
Alcohol 3 or more units per day	380 (31.1 %)	174 (31.8 %)	72 (36.0 %)	95 (35.6 %)	38 (20.3 %)

Using the FRAX model, the median and mean 10-year probability of a major osteoporotic fracture according to the CRFs alone was 7.9 % (8.8 ± 4.3 %), while the 10-year probability of hip fracture risk was 2.7 % (3.5 ± 3.1 %). Forty-nine patients (4.0 %) had a major osteoporotic fracture risk of more than 20 % and 643 patients (52.7 %) had a hip fracture risk of more than 3 %. A comparison between the ADT monotherapy (*n* = 187) and non-ADT (*n* = 399) groups showed that the major osteoporotic fracture risk > 20 % and hip fracture risk > 3 % was significantly higher in the ADT group than in the non-ADT group (ADT: 10 (5.3 %) and 118 (63.1 %), non-ADT 13 (3.3 %) and 189 (47.4 %), *p* < 0.001 and *p* < 0.001, respectively).

**Fig. 1:**
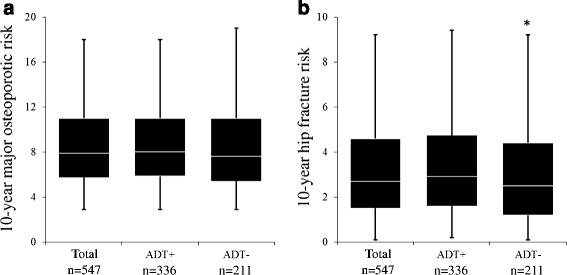
The FRAX score with or without ADT in the patients who received brachytherapy. The FRAX score between prostate cancer patients who received brachytherapy with ADT and those without ADT was compared. **a** According to the 10-year major osteoporotic risk, the ADT group showed a tendency toward a higher FRAX score (*p* = 0.12). **b** According to the 10-year hip fracture risk, the ADT group showed a significantly higher FRAX score than the non-ADT group (*p* = 0.04)

The major osteoporotic risk of patients who received periodical ADT in combination with brachytherapy tended to be higher than that of the patients who did not receive ADT (*p* = 0.12). On the other hand, the hip fracture risk of the patients who received ADT in combination with brachytherapy was significantly higher than that of the patients who did not receive ADT (*p* = 0.04) (Fig. 1).

**Fig. 2:**
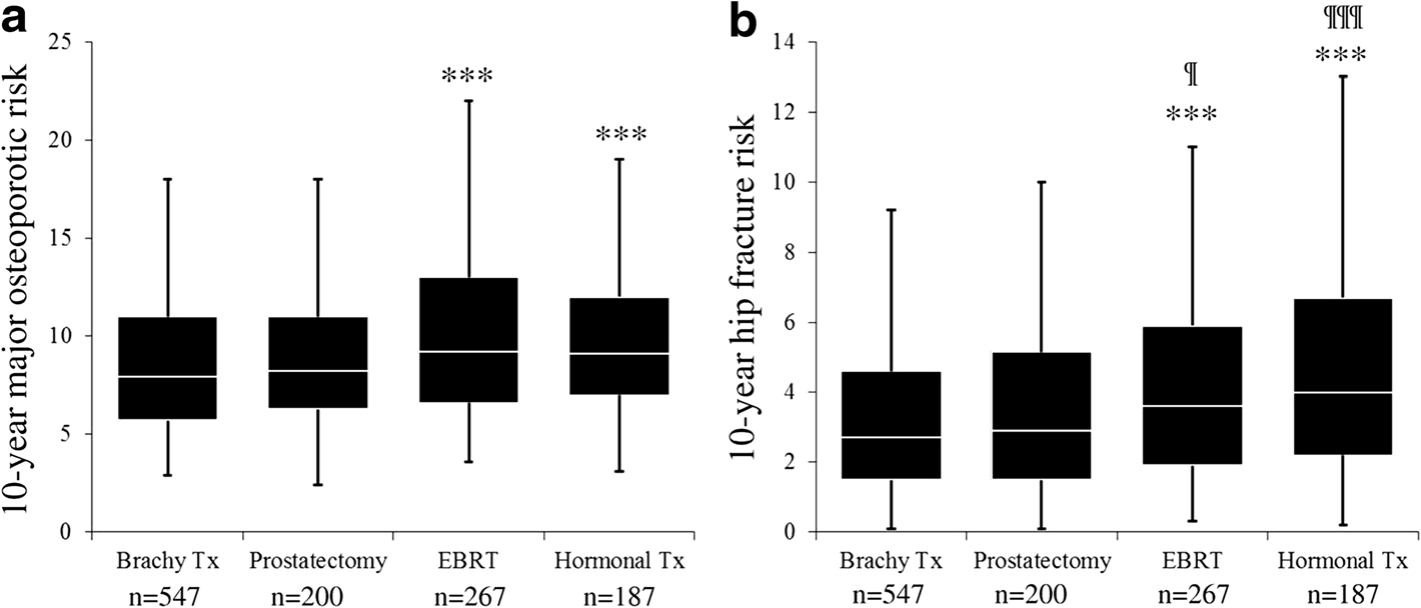
The FRAX score among various prostate cancer therapies. **a**: The 10-year major osteoporotic risk. The EBRT and ADT monotherapy groups showed significantly higher FRAX scores than the brachytherapy group (*p* < 0.001, *p* < 0.001, respectively). **b**: The 10-year hip fracture risk. The EBRT and ADT monotherapy groups showed higher FRAX scores than both the brachytherapy group (*p* < 0.001, *p* < 0.001, respectively) and the prostatectomy group (*p* < 0.05, *p* < 0.001, respectively)

Regarding the therapies for prostate cancer, the major osteoporotic risk was 7.9 % (8.8 ± 4.3 %) in the brachytherapy group, 8.2 % (9.3 ± 5.2 %) in the radical prostatectomy group, 9.2 % (19.2 ± 5.0) in the EBRT group, and 9.1 % (10.4 ± 5.4 %) in the ADT group. The EBRT and ADT monotherapy groups showed significantly higher major osteoporotic risk than the brachytherapy group (*p* < 0.001 and *p* < 0.001, respectively). The hip fracture risk was 2.7 % (3.5 ± 3.1 %) in the brachytherapy group, 2.9 % (3.7 ± 3.3 %) in the radical prostatectomy group, 3.6 % (4.5 ± 4.0 %) in the EBRT group, and 4.0 % (5.2 ± 4.6 %) in the ADT group. The EBRT and ADT groups showed significantly higher hip fracture risk than both the brachytherapy and prostatectomy groups (*p* < 0.001, *p* < 0.001 and *p* = 0.02, *p* < 0.001, respectively) (Fig. 2).

The major osteoporotic risk > 20 % and hip fracture risk > 3 % were 2.7 and 46.6 % in the brachytherapy group, 3.5 and 48.5 % in the radical prostatectomy group, 5.6 and 60.7 % in the EBRT group, and 5.9 and 70.1 % in the ADT group.

**Fig. 3:**
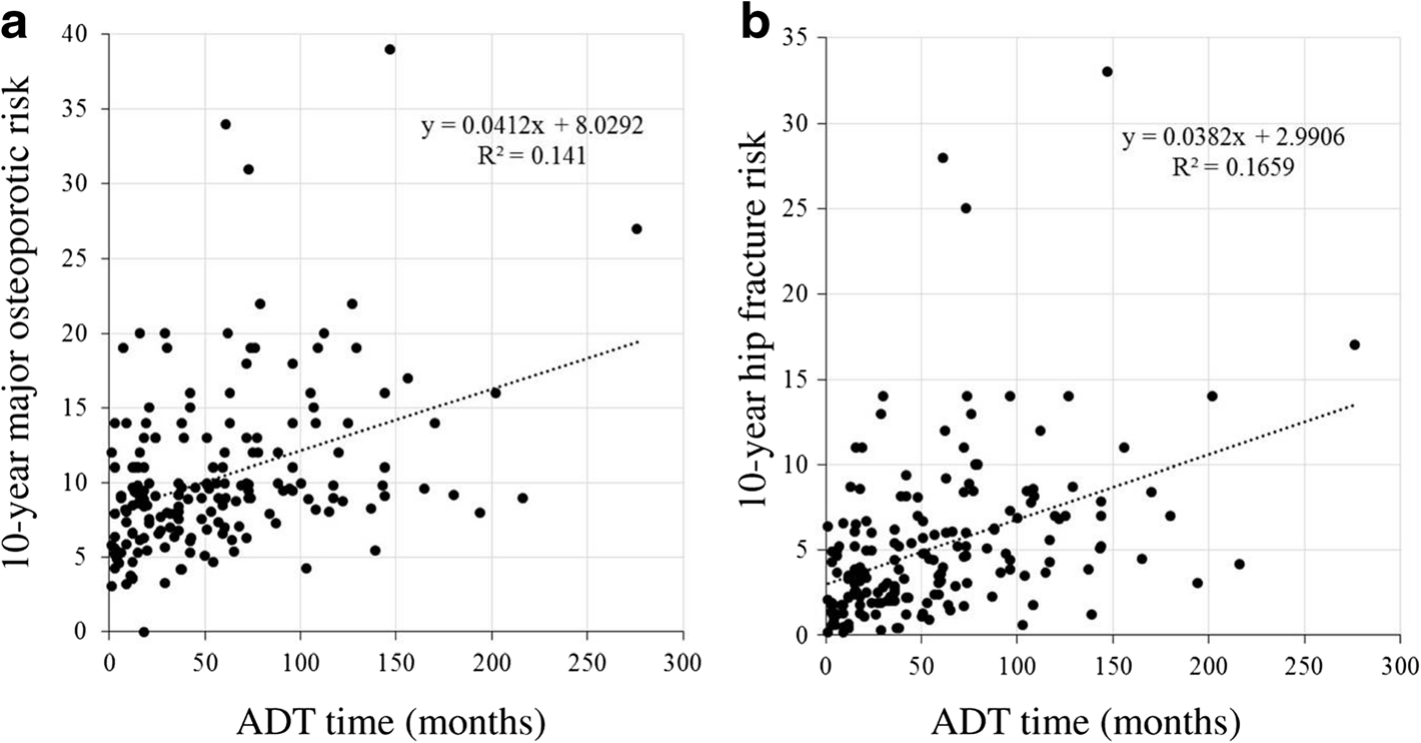
The FRAX score correlated with the duration of ADT. **a**: The 10-year major osteoporotic risk was positively correlated with the duration of ADT (R^2^ = 0.141, *p* < 0.0001). **b**: The 10-year hip fracture risk was positively correlated with the duration of ADT (R^2^ = 0.1659, *p* <0.0001)

When analyzed in the ADT monotherapy group, the duration of ADT correlated with both the major osteoporotic risk and hip fracture risk (R^2^ = 0.141, *p* < 0.001 and R^2^ = 0.166, *p* < 0.001, respectively) (Fig. 3).

## Discussion

This study revealed that ADT increased the FRAX score and that the duration of ADT correlated with the FRAX score. In particular for prostate cancer patients treated with hormonal therapy, fractures are a major complication which can influence both the activities of daily life and life expectancy. Although the median 10-year probability of major osteoporotic and hip fracture risks were not higher than expected, 7.9 and 2.7 %, respectively, ADT elevated those probabilities as expected. It is plausible that patients who received ADT were comparatively older than those who received brachytherapy or RRP. We found that the FRAX score correlated with the duration of ADT. In most of the patients in the brachytherapy-treated group, ADT was performed within one year. Consequently, there were no large differences occurred within the brachytherapy group regardless of whether the patients received ADT or not. ADT, a common treatment option for patients with prostate cancer that reduces circulating testosterone levels, has a detrimental effect on BMD, leading to a substantial increase in the fracture risk [8, 15, 16]. Men undergoing ADT are four times more likely to develop significant bone deficiency [8, 15]. ADT is known to cause a decrease in BMD, and therefore patients who receive ADT should be assumed to have secondary osteoporosis when calculating the fracture risk using the FRAX score [2, 11]. It has been well established that fractures are associated with significant morbidity and mortality. This study showed that the increase in FRAX score by ADT correlated with the duration of ADT. During ADT, it might be recommended that patients, especially those who have received ADT for longer periods of time, perform regular muscle-strengthening exercises or increase their vitamin D intake [17].

Although ADT use was positively correlated with the FRAX score (R^2^ = 0.141), the correlation was not so high. We speculate that the relatively weak correlation resulted from the use of the FRAX score for assessment rather than the DEXA score. We are presently conducting another study to reveal the correlation between the ADT time and the risk of fracture using the DEXA score and some bone related biomarkers. Although there were some indications for intervention to prevent fracture, we set the cut-off point as 20 % in patients with major osteoporosis and 3 % in patients with hip fracture because a 10-year probability of major osteoporotic risk >20 % and hip fracture >3 % is considered to represent a clinically-relevant degree of risk [11] according to the American National Osteoporosis Foundation Clinician’s Guide to Prevention and Treatment of Osteoporosis. Our study also found that the ADT group showed a significantly higher fracture risk, which requires fracture prevention.

There are two limitations associated with the present study. First, we assessed the FRAX score and did not determine the patients’ real fracture incidences over the 10-year follow-up period. The FRAX was developed by the WHO to predict the fracture risk; however, this score has some ethnicity-related differences, although there are algorithms available for various ethnic groups. In addition, this score is typically used during the clinical courses. Therefore, assessing the fracture risk using the FRAX score is valuable. Second, we did not include the BMD score in this study. BMD is a useful tool to predict fractures and initiate the intervention for bone management [12, 13]. The National Osteoporosis Foundation and other groups have recommended guidelines for BMD testing according to clinical factors. Additionally, the FRAX score may be used with or without BMD. In the present study we did not use BMD testing in our assessment of the FRAX score. In several cases, we also performed dual-energy X-ray absorptiometry (DEXA) to calculate the BMD and found that the FRAX score was reduced when we used BMD (data not shown). In the present study FRAX was therefore used as a screening tool to determine whether or not BMD testing should be performed. The 10-year probability of a major osteoporotic fracture increases when the femur neck T-scores are added to the CRFs in the FRAX algorithm, and this population has a high fracture probability even in the absence of CRFs [18, 19]. However, in clinical use, not all patients undergo DEXA due to the medical costs. Accordingly, we recommend the use of the FRAX score for prostate cancer patients, especially those treated with ADT, as a screening tool in addition to bone management therapy. The FRAX algorithm without the BMD values was superior to BMD alone in identifying the patients at a higher risk of fractures. Therefore, patients with high FRAX scores should receive DEXA in order to avoid additional bone management therapy.

## Conclusion

The duration of ADT can influence the FRAX score and the ADT group showed a significantly higher FRAX score and an increased need for additional bone management treatment. This large study is needed to show that ADT influenced the FRAX scores.

## Abbreviations

ADT, Androgen deprivation therapy; FRAX, fracture risk assessment tool; BMD, bone mineral density; DEXA, dual-energy X-ray absorptiometry; CRF, clinical risk factors; RRP, radical retropubic prostatectomy
